# A Multimodal Mobile Sleep Intervention for Young Adults Engaged in Risky Drinking: Protocol for a Randomized Controlled Trial

**DOI:** 10.2196/26557

**Published:** 2021-02-26

**Authors:** Lisa M Fucito, Garrett I Ash, Kelly S DeMartini, Brian Pittman, Nancy P Barnett, Chiang-Shan R Li, Nancy S Redeker, Stephanie S O'Malley

**Affiliations:** 1 Department of Psychiatry Yale University School of Medicine New Haven, CT United States; 2 Yale Cancer Center New Haven, CT United States; 3 Smilow Cancer Hospital Yale-New Haven Hospital New Haven, CT United States; 4 Pain, Research, Informatics, Medical Comorbidities and Education Center Veterans Affairs Connecticut Healthcare System West Haven, CT United States; 5 Yale University School of Public Health New Haven, CT United States; 6 Department of Behavioral and Social Sciences School of Public Health Brown University Providence, RI United States; 7 Yale University School of Nursing Orange, CT United States

**Keywords:** sleep, binge drinking, young adults, mHealth, biosensor, behavior therapy, mobile phone

## Abstract

**Background:**

This paper describes the research protocol for a randomized controlled trial of a multimodal mobile sleep intervention for heavy-drinking young adults. Young adults report the highest rates of heavy, risky alcohol consumption and are a priority population for alcohol prevention and intervention efforts. Alcohol strategies that leverage other health concerns and use technology may offer an innovative solution. Poor sleep is common among young adults and is a risk factor for developing an alcohol use disorder. Moreover, young adults are interested in information to help them sleep better, and behavioral sleep interventions address alcohol use as a standard practice.

**Objective:**

The primary aim of this study is to assess the effectiveness of a 2-week multimodal mobile sleep intervention for reducing drinks consumed per week among heavy-drinking young adults. We will explore the effects on alcohol-related consequences, assessing quantitative and qualitative sleep characteristics as secondary aims. The study’s goals are to identify the optimal combination of sleep intervention components for improving drinking outcomes, the feasibility and acceptability of these components, and the potential mechanisms by which these components may promote alcohol behavior change.

**Methods:**

Young adults (aged 18-25 years) who report recent heavy drinking will be randomly assigned to one of three conditions: mobile sleep hygiene advice (n=30), mobile sleep hygiene advice and sleep and alcohol diary self-monitoring (n=30), or mobile sleep hygiene advice, sleep and alcohol diary self-monitoring, and sleep and alcohol data feedback (n=60). For the feedback component, participants will complete two web-based sessions with a health coach during which they will receive summaries of their sleep and alcohol data, and the potential association between them along with brief advice tailored to their data. All participants will wear sleep and alcohol biosensors daily for 2 weeks for objective assessments of these outcomes.

**Results:**

The study was funded by the National Institutes of Health in May 2018. Recruitment began in December 2018 and will be concluded in Spring 2021. As of February 4, 2021, we have enrolled 110 participants.

**Conclusions:**

Ultimately, this research could result in an efficacious, low-cost intervention with broad population reach through the use of technology. In addition, this intervention may substantially impact public health by reducing alcohol use disorder risk at a crucial developmental stage.

**Trial Registration:**

ClinicalTrials.gov NCT03658954; https://clinicaltrials.gov/ct2/show/NCT03658954

**International Registered Report Identifier (IRRID):**

DERR1-10.2196/26557

## Introduction

### Background

#### Alcohol Use and Sleep Among Young People

Alcohol use disorder (AUD) onset peaks during young adulthood (ie, 18-25 years) [1]. Compared with older adults, young adults report more frequent and heavier alcohol use, which is linked to substantial negative consequences, including the risk of accidental injury, the primary cause of death among young adults [2-4]. Current alcohol interventions for young adults have modest effects [5-7], and young adults rarely self-identify for specialized alcohol treatment [8,9]. Thus, more work is needed to identify effective alcohol interventions and novel treatment engagement strategies to reduce this substantial public health burden.

A novel approach is to target poor sleep, a common complaint among young adults who drink heavily [10,11] and an AUD risk factor in young adulthood [12-18]. Sleep problems in young adults may be because of developmental sleep changes that begin with puberty and continue into young adulthood. During this developmental period, there is a need for more sleep [19] and a preference for later bed and wake times [20], which often conflict with school or work demands and social or cultural obligations [21]. To cope with these conflicts, adolescents and young adults may maintain shorter, more variable sleep schedules putting them at risk for sleep problems, excessive daytime sleepiness, and other negative consequences [21-23].

AUD risk is an important correlation and a potential consequence of poor sleep. In young adults, greater alcohol consumption and alcohol-related consequences are associated with shorter sleep duration, poorer sleep quality, and more delayed bed or wake times [10,11]. In addition, various sleep problems in adolescence predict earlier AUD onset and a greater risk of heavy drinking, alcohol-related consequences, and AUD in young adulthood [12,18,24]. Furthermore, poor sleep in young adults predicts a greater future risk of alcohol-related problems [14].

The nature of this sleep-alcohol association in young adults is not clear but is likely bidirectional. Heavy alcohol use may directly disrupt sleep [25-28]. Conversely, vulnerability to both poor sleep and heavy drinking may be because of mental health concerns [29]. Another possibility is that poor sleep may reduce control to resist drinking or alter reward sensitivity through adverse cognitive function effects [21]. In neuroimaging studies, healthy adolescents with poor sleep habits exhibited altered reward processing and reduced cognitive control compared with adolescents with good sleep patterns [21,30-32]; sleep-deprived adults also exhibited an altered reward processing [33-35]. It is also possible that poor sleep is associated with disrupted chronobiology that interrelates a host of neuroendocrine and physiological changes and alcohol misuse [36,37]. Regardless of the initial cause, poor sleep and heavy drinking likely become a negative feedback cycle that both interact and influence each other. Thus, an important hypothesis that warrants further investigation is whether improving sleep might reduce alcohol-related risks among young adults.

#### Sleep Interventions to Reduce Heavy Drinking

With the exception of our preliminary work [38] and a recent pilot study [39], sleep interventions for reducing drinking and alcohol-related risks have only been tested in older adults and focused on cognitive behavioral therapy for insomnia [40,41]. In older populations, poor sleep is a well-established alcohol relapse risk factor [42-45], but sleep interventions have yielded mixed results [28,42,46]. Among older adults, chronic AUDs may cause permanent sleep changes that are not amenable to sleep interventions [45]. Conversely, in young adults who drink heavily, sleep problems may be reversible because of other factors that are amenable to treatment (eg, poor sleep hygiene). Early intervention in young adults may prevent the establishment of persistent sleep problems and continuous heavy drinking.

Another potential advantage of sleep interventions is that heavy-drinking young adults are open to information to help them sleep better [47] and standard-of-care sleep interventions address alcohol use [48]. Specifically, individuals are advised to moderate alcohol use for better sleep and are informed of the sleep-disruptive effects of alcohol [48]. Thus, sleep interventions may provide a potential gateway for intervening in alcohol use and engaging heavy-drinking young adults in treatment. This novel engagement strategy could potentially benefit this population, as it does not rely on self-identification for alcohol treatment.

### Objectives

#### Formative Research

To test poor sleep as a novel treatment target, we conducted the first preliminary test of a sleep intervention in 42 heavy-drinking young adults with sleep concerns [38]. We intentionally targeted sleep more broadly than the specific problem of insomnia. Poor sleep in young adults, especially college students, manifests in several ways, such as sleep deprivation or restriction, delayed sleep phase syndrome, or insomnia [49-51]. Young adults face unique pressures (eg, college life) and many report voluntarily altering their sleep schedules to meet them [49,52]. Our goal was to develop a sleep intervention that could engage and benefit a larger proportion of young adults who have sleep concerns and engage in risky drinking than just those with insomnia. We derived the sleep intervention from previous evidence-based interventions for improving sleep and drinking in young adults and formative work in this population [38,53]. The study, advertised through social media, targeted heavy-drinking young adults with sleep concerns and generated high interest (ie, 400+ inquiries in 4 months). Eligible participants were randomly assigned to one of two 4-week web-based conditions: (1) a sleep intervention that included a brief alcohol intervention or (2) a general wellness active control intervention with minimal sleep and alcohol advice. Consistent with our hypotheses, greater sleep improvement predicted less drinking (regardless of participants’ sleep concern level). However, contrary to expectations, participants in both conditions had medium-to-large improvements in alcohol and sleep outcomes. The effects on alcohol consumption were larger than those of typical brief alcohol intervention studies for young adults [5-7].

These results generated new hypotheses and directions for further sleep intervention refinement. The unexpected finding of comparable improvements across both conditions suggested that common elements may have contributed to the outcomes. Participants in both conditions received brief sleep hygiene advice and advice to moderate their drinking for improved sleep. This brief advice alone may have been sufficient to improve sleep and reduce drinking. In addition, participants in both conditions actively monitored their sleep and alcohol use. Sleep hygiene education is effective for improving sleep in young adults [50,54]. Similarly, self-monitoring (SM) can improve many health behaviors such as poor sleep and alcohol use [55-59], as it may help individuals learn more about their behavior, identify discrepancies between their goals and behavior, and acquire a greater sense of control over their behavior [60,61]. According to the Theory of Planned Behavior (TPB), perceived behavioral control is a factor that can increase intentions to change behavior [62]. To clarify whether sleep SM, including monitoring of drinking, is an effective intervention component, a control condition that does not include SM is needed in a follow-up study.

Our qualitative research also yielded insights into ways to improve our intervention. Specifically, participants expressed in exit interviews a desire to receive (1) personalized feedback about their sleep data and the links with alcohol use and (2) health advice tailored to this data. Health feedback, another effective behavior change strategy in line with the TPB [62], may facilitate behavior awareness and goal setting; ongoing feedback may reinforce behavior change, increase motivation, and enhance self-efficacy [61]. Greater positive beliefs about the outcome of behavior change and greater confidence in one’s ability to perform this behavior may increase behavior change intentions [62].

#### Current Protocol

To our knowledge, no studies have tested these 3 sleep intervention components in combination (ie, SM, evidence-based sleep intervention content, and personalized sleep or alcohol feedback) for alcohol prevention or early intervention. This approach, delivered through a mobile platform, aligns well with the help-seeking behaviors of heavy-drinking young adults and their comfort and facility with technology. Many young adults do not perceive a need for help with their drinking and are increasingly less likely to visit a health care provider [63,64]. Thus, other *on ramps* to alcohol preventive services are urgently needed. Young adults are concerned about sleep and health [47]. Therefore, it may be useful for this population to embed alcohol-related content within other health programs and connect alcohol use to health outcomes. Young adults are also the largest consumers of new health technology [65]. Within the last decade, there has also been an explosion in the importance of sleep [66-68] and technology options for improving it (eg, mobile apps, wearable sleep biosensors) [69,70]. This paper describes the rationale and design of a randomized controlled trial to develop and test a multimodal mobile sleep intervention to reduce alcohol use and alcohol-related consequences among young adults who engage in risky drinking.

## Methods

### Design

Heavy-drinking young adults aged 18 to 25 years (N=120) will be randomized using a 1:1:2 ratio to one of 3 conditions: (1) mobile sleep hygiene *advice* (*A*; n=30), (2) mobile sleep hygiene *advice* and sleep and alcohol diary *SM* (*A+SM*; n=30), or (3) mobile sleep hygiene *advice*, sleep and alcohol diary *SM*, and sleep and alcohol data *feedback* (*A+SM+F*; n=60); 5 pilot participants will first be tested in the A+SM+F condition to finalize the study procedures and refine the feedback reports. We added more participants to A+SM+F to have a larger sample to assess the variability of participants’ sleep and alcohol data and the perceived acceptability and helpfulness of data feedback and advice tailored to this data for heavy-drinking young adults.

Our primary hypothesis is that combining all 3 intervention components (ie, A+SM+F) will yield the greatest reductions in alcohol consumption and alcohol-related consequences compared with combining advice with active SM (A+SM) or providing brief advice alone (A), in that order. We also anticipate that the 3-component intervention (A+SM+F) will rank best among participants and result in the largest improvements in quantitative and qualitative sleep outcomes. These hypotheses are based on our preliminary research findings and behavior change theory [62]. The 3-component intervention targets most behavior determinants, including perceived behavioral control, beliefs and attitudes about behavior, and motivation. We will explore whether sleep intervention promotes reductions in drinking through behavioral control changes and/or changes in attitudes or perceptions about alcohol.

**Figure 1:**
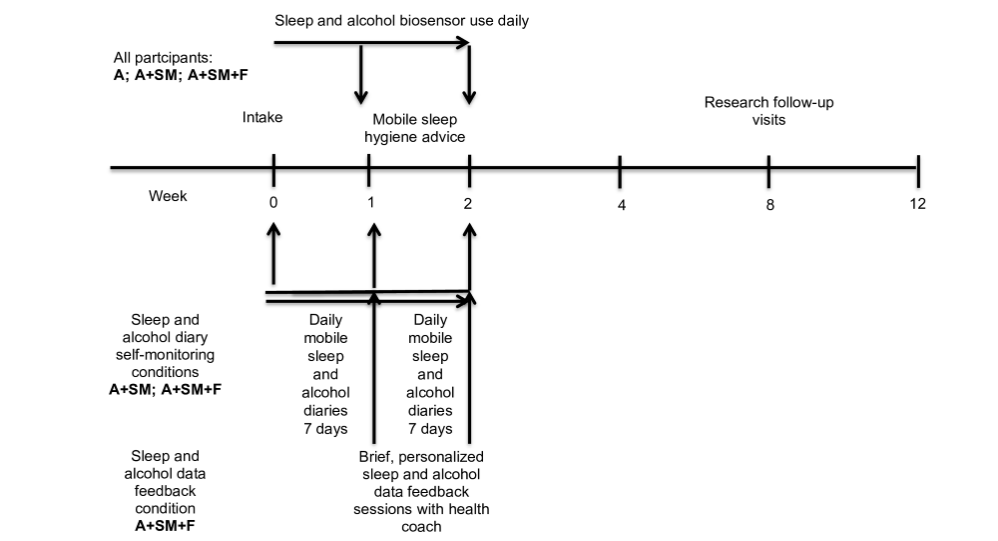
Single participant timeline.

Following intake, all participants will wear mobile sleep and alcohol biosensors daily for 2 weeks to measure sleep and alcohol outcomes. However, participants will not receive immediate feedback from these devices. A+SM and A+SM+F participants will also complete daily mobile sleep and alcohol diaries during the 2-week period but will not receive immediate feedback on this diary data. Once a week, all participants will receive brief mobile sleep hygiene advice using the program from our pilot study. In the A+SM+F condition, participants will also have brief sessions with a health coach once a week to review their sleep and alcohol diary and biosensor data and the potential bidirectional links between them, along with brief advice tailored to these data. Participants will receive an electronic copy of their health feedback after each session. All participants will complete follow-ups at weeks 4, 8, and 12 (see Figure 1 for a single participant flowchart).

### Participants

Young adults will be included in the study if they (1) are aged between 18 and 25 years, (2) report ≥3 heavy-drinking occasions in the past 2 weeks (ie, ≥5 drinks on 1 occasion for men; ≥4 for women), (3) report sleep concerns, (4) are willing or able to complete daily mobile diaries and wear sleep or alcohol biosensors, (5) report Alcohol Use Disorders Identification Test–Consumption (AUDIT-C) scores indicative of a risk of drinking harm (ie, ≥7 for men; ≥5 for women) [71], (5) are English speaking, and (6) have a smartphone for syncing biosensor data. An estimated 86% of young adults own a smartphone [72].

Young adults will be excluded if they (1) have a history of a sleep disorder or a severe alcohol use disorder (ie, severe alcohol withdrawal syndrome); (2) are currently enrolled in alcohol or sleep treatment; (3) report night or rotating shift work or travel beyond 2 time zones in the month before and/or plan to travel beyond 2 time zones during study participation; (4) exhibit current, severe psychiatric illness by history or examination; (5) have medical conditions contraindicated for the use of the ankle-worn alcohol biosensor (ie, circulation problems, neuropathy, deep vein thrombosis, leg ulcers, tendonitis, diabetes, pregnancy, history of swelling, nickel or other metal allergies, pacemaker, or any other implanted medical device); or (6) meet current Diagnostic and Statistical Manual of Mental Disorders, fifth edition (DSM-V), substance use disorder criteria for substances other than marijuana or have a positive urine drug screen for opiates, cocaine, barbiturates, benzodiazepines, amphetamines, or phencyclidine. Marijuana use is very common among heavy-drinking young adults [73]; exclusion limits recruitment and external validity.

### Procedures

#### Screening and Randomization

We will recruit most participants through web advertising or social media (eg, Facebook, Instagram, Snapchat), a method we have successfully used to recruit young adults and heavy drinkers [74,75]. We will also display notices around the local community. Interested individuals who contact investigators by telephone or email will be directed to a secure website link to complete a brief, 5-min prescreening survey. Digital advertisements will also direct volunteers to the prescreener. Before completing the prescreener, volunteers must provide informed consent. Following completion of the prescreener, research staff will contact potential participants and inform them of their initial eligibility status.

Individuals who meet initial screening criteria will complete an in-person intake to determine final study eligibility and receive US $30. Eligible participants will complete baseline questionnaires and be randomized to their condition. A statistician will create a randomization list that will be stratified by sex and implemented through REDcap, an electronic clinical trial management system, to ensure allocation concealment.

#### 2-Week Treatment Phase

Immediately following randomization, participants will begin the 2-week intervention period that will vary by condition assignment (see Table 1 for a comparison of study conditions). At the end of week 2, all participants will complete an exit interview and a survey to assess the acceptability of the study intervention components they received.

**Table 1:** Intervention conditions.

Condition	Intervention components			
	Daily sleep and alcohol biosensor use	Mobile sleep hygiene advice (includes brief alcohol advice)	Daily sleep and alcohol diary self-monitoring	Personalized sleep and alcohol feedback sessions with a coach
A^a^ (n=30)	✓^b^	✓	—^c^	—
A+SM^d^ (n=30)	✓	✓	✓	—
A+SM+F^e^ (n=60)	✓	✓	✓	✓

^a^A: advice.

^b^Intervention component present in intervention condition.

^c^Intervention component not present in intervention condition.

^d^SM: self-monitoring.

^e^F: feedback.

##### Biosensors

Participants in all conditions will wear sleep and alcohol biosensors daily for 14 days. The research coordinator will fit participants with biosensors at intake and then arrange brief weekly visits with participants to synchronize their devices to the study computer and download their data. To measure objective quantitative sleep characteristics, participants will wear a Philips Respironics Actiwatch Spectrum Plus actigraph device, a well-validated wrist-worn sleep biosensor that measures sleep or wake activity. Participants will be instructed to continuously wear the waterproof Actiwatch on their nondominant wrist and depress the event marker when ready to initiate sleep after getting into bed and immediately upon waking to indicate the end of the sleep episode. Actigraphy is a valid, reliable methodology to objectively estimate sleep based on measuring activity and inactivity and is sensitive to changes over time and interventions [76]. As an objective measure of alcohol use, participants will wear the secure continuous remote alcohol monitor (SCRAM) ankle bracelet from Alcohol Monitoring Systems, Inc. The SCRAM uses an electrochemical sensor to sample transdermal alcohol concentration (TAC) levels from sweat in the skin at regular intervals (ie, every 30 min) and store readings for later download [77]. SCRAM TAC readings are highly correlated with peak blood alcohol concentrations (BACs) and self-reported alcohol use [77]. The device is particularly effective at detecting heavy alcohol consumption (ie, ≥5 drinks) [77]. The SCRAM is water resistant (participants have to avoid swimming or baths) and can only be removed by cutting the strap. To encourage adherence, participants will be compensated for wearing the devices each day (US $2 per day for a total of US $28) and for returning them (US $10 per device for a total of US $20). Participants will also wear a new, wrist-worn transdermal alcohol biosensor, BACTrack Skyn, and will receive US $1 per day for wearing it (total US $14). Unlike the large SCRAM biosensor, which relies on active airflow, the smaller Skyn biosensor relies on passive airflow and permits more regular TAC sampling (ie, every 20 seconds) [78]. Preliminary evidence from controlled laboratory studies suggests that Skyn is sensitive to alcohol consumption changes [78].

##### Mobile Sleep Hygiene Advice

All participants will receive brief sleep hygiene advice via a mobile sleep program adapted from our pilot study. Module 1, which they will view on the day of the first biosensor data download (middle of week 1), focuses on behaviors that affect sleep (ie, exercise, eating, stimulant use, marijuana use, use of sleep aids, alcohol use, and sleep routines) and general recommendations for optimal sleep. Module 1 also contains a brief alcohol intervention content (ie, standard drink conventions and moderate drinking guidelines, how to calculate blood alcohol level and the effects at different levels, and normative information about young adults’ drinking, controlled drinking strategies, effects of alcohol on sleep, and advice to moderate drinking for improved sleep). Module 2, which they will view on the day of the second biosensor data download, focuses on establishing a good sleep environment (ie, optimal lighting, temperature, noise, comfort, and stress levels). Both modules are approximately 10 min in length. Biosensor data are not viewed anywhere in this mobile sleep hygiene advice program; rather, they are saved for the health feedback coaching sessions discussed below and thus are only visible to participants in A+SM+F.

##### Mobile Sleep and Alcohol Diaries

Participants in the A+SM and A+SM+F conditions will complete mobile diaries of their sleep and alcohol use daily for 14 days. Diaries will be programmed in Qualtrics and sent to participants each morning via text message. To encourage adherence, participants will be compensated for completing diaries (US $1 per day for a total of US $14).

##### Health Feedback Coaching Sessions

**Figure 2:**
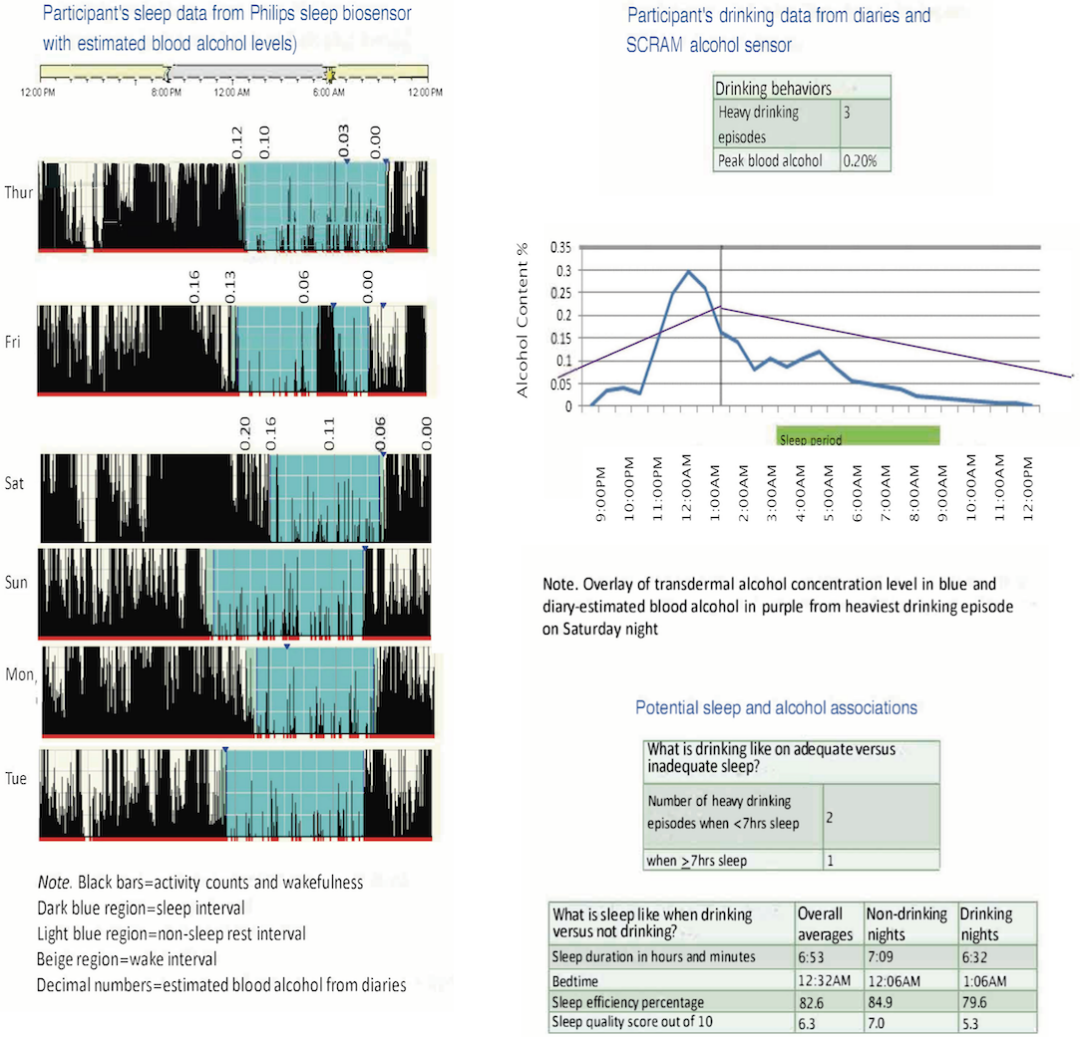
Sample participant feedback. SCRAM: secure continuous remote alcohol monitor.

Participants in A+SM+F will have 2 weekly, brief sessions with a health coach to review their health data and receive brief health advice. Participants will receive the following information in handout form: (1) 7-day average quantitative sleep characteristics from the Actiwatch; (2) 7-day average of qualitative sleep diary ratings; (3) diary entries of alcohol consumption (ie, total drinks consumed, number of heavy-drinking occasions, maximum drinks on an occasion) and tobacco and marijuana use (ie, total occasions); (4) estimated BAC levels from the diaries and estimated TAC levels from the SCRAM; (5) average quantitative and qualitative sleep characteristics on drinking occasions versus nondrinking occasions; (6) total occasions of alcohol, tobacco, and/or marijuana use on days following ≥7 hours of sleep compared with <7 hours of sleep (ie, the minimum recommendation for young adults); and (7) health recommendations for sleep (ie, optimal duration, timing, consistency, efficiency) and alcohol use for young adults (ie, moderate drinking guidelines). Participants will also receive the visual output data from the Actiwatch with their estimated BAC level superimposed over their sleep window for any drinking occasions that occurred close to bedtime (see Figure 2 for a sample feedback handout). Similarly, they will receive visual representations of their BAC and TAC data curves with their sleep window superimposed (Figure 2). These figures will enable participants to understand how long it takes alcohol to metabolize, how high alcohol levels may remain while they are sleeping and upon waking, and how their sleep quantitative and qualitative data may vary between drinking and nondrinking occasions (eg, wakefulness during sleep, perceived sleepiness upon waking). In accordance with motivational enhancement therapy for problem drinkers, the health coach will review the health feedback with participants and engage them in an open-ended, empathic, nonjudgmental discussion in an effort to enhance their motivation to change both behaviors [79]. The health coach will encourage a discussion of future plans regarding both behaviors, offer respectful advice to change risky sleep and drinking behaviors, and offer a menu of change strategies tailored to participants’ responses and health data (eg, maintaining a regular sleep schedule, setting a minimum sleep duration threshold of 7-9 hours, using scheduled napping to reduce sleep deprivation effects, shifting alcohol use earlier in the evening, lowering peak BAC before bedtime, following moderate drinking guidelines, controlled drinking strategies).

#### Follow-Up

At weeks 4, 8, and 12, participants will complete follow-up visits to assess potential changes in primary and secondary outcomes (see Table 2 for the schedule of assessments). Participants will be compensated for completing the week 4, 8, and 12 visits in escalating amounts to encourage adherence (ie, US $50, US $55, US $60).

**Table 2:** Schedule of assessments.

Variables and assessments			Intake		Diary		Alcohol and sleep biosensors		Weeks 1, 2, 4, 8, and 12
**Eligibility**									
	Demographics	✓^a^		—^b^		—		—	
	DSM-V^c^ diagnoses	✓		—		—		—	
	Urine drug screen and breath alcohol	✓		—		—		✓	
	AUDIT^d^	✓		—		—		—	
	Endorse sleep concerns	✓		—		—		—	
**Alcohol variables**									
	Timeline followback	✓		—		—		✓	
	Alcohol-related consequences	✓		—		—		4, 8, 12	
	SCRAM^e^ and TAC^f^ levels	—		—		✓		—	
	Alcohol diary	—		✓		—		—	
**Sleep variables**									
	PROMIS^g^ sleep-related impairment	✓		—		—		4, 8, 12	
	PROMIS sleep disturbance	✓		—		—		4, 8, 12	
	Pittsburgh sleep diary	—		✓		—		—	
	Positive and negative affect	✓		—		—		4, 8, 12	
	Chronotype and morningness or eveningness	✓		—		—		—	
	Actiwatch: duration; bed time or wake time; min awake after sleep onset; sleep efficiency	—		—		✓		—	
**Mechanisms**									
	TPB^h^ questionnaires; cognitive tasks	✓		—		—		4	
**Feasibility**									
	Adherence (diaries, biosensors, and tips)	—		✓		✓		✓	
**Acceptability**									
	Treatment evaluation survey; exit interview	—		—		—		2	

^a^Variables or assessments administered at this time point.

^b^Variables or assessments not administered at this time point.

^c^DSM-V: Diagnostic and Statistical Manual of Mental Disorders, fifth edition.

^d^AUDIT: Alcohol Use Disorders Identification Test–consumption.

^e^SCRAM: secure continuous remote alcohol monitor.

^f^TAC: transdermal alcohol concentration.

^g^PROMIS: patient-reported outcomes measurement information system.

^h^TPB: Theory of Planned Behavior.

### Variables and Measures

#### Eligibility

Interviews, questionnaires, and biosamples will be used to verify participant eligibility. These measures will include (1) a sociodemographic survey; (2) the Structured Clinical Interview for DSM-V [80] (ie, current and past substance use disorders, other current psychiatric diagnoses); (3) the Alcohol Use Disorders Identification Test, a reliable, valid alcohol use screener (at-risk drinking eligibility will be based on recommended AUDIT-C cut-off scores for young adults) [71]; (4) a single-item question to assess whether participants were concerned about their sleep using a dichotomous item (ie, yes or no) used in our previous research [38]; (5) a urine toxicology test kit for opiates, cocaine, barbiturates, amphetamines, benzodiazepines, or phencyclidine (JANT Pharmaceuticals); and (6) a breath alcohol concentration test using a hand-held breathalyzer unit—an Alcohol-Sensor III (Intoximeter Inc). Participants need to test negative to provide consent at intake and need to test <0.04% at subsequent in-person treatment and assessment visits.

#### Alcohol Variables

We will administer the Timeline Followback Interview, a standardized, validated, and reliable experimenter-administered interview to obtain daily reports of drinking that will be used to compute summary measures of alcohol use (ie, total drinks, drinks per day, drinks per drinking day) for the 30-day period before study enrollment and monthly following intake for a total of 4 months [81]. Calendar prompts and memory aids (eg, holidays) are used to facilitate accurate recall of substance use during the targeted period. Participants will complete the Young Adult Alcohol Consequences Questionnaire, a reliable, valid survey of 48 consequences of alcohol consumption predictive of drinking persistence among young adults [82]. We will derive estimates of participants’ peak and average TAC levels from the SCRAM biosensor. Alcohol use episodes will be detected using the criteria developed by Barnett et al [83] and using software that processes the sensor data accordingly [84]. In the A+SM and A+SM+F conditions, participants will complete the Drinking Self-Monitoring Log, a standardized, validated methodology for measuring drinking on a daily or drink-by-drink basis [85]. Participants will record the total number of *standard drinks* consumed the preceding day, the type of beverages consumed, and the duration of alcohol consumption to allow for BAC level estimates. Diaries will also assess alcohol cravings and the drinking context.

#### Sleep and Sleep-Related Characteristics

We will use multiple assessments to characterize participants’ quantitative and qualitative sleep characteristics and the potential consequences of their sleep. All participants will wear an Actiwatch. Actigraphy is a valid and reliable methodology used in research to objectively estimate sleep or wake activity. Validation studies provide evidence of its reliability and validity relative to well-validated ambulatory and laboratory sleep assessment methods (ie, polysomnography) [86-88]. We will derive the following quantitative sleep variables: sleep onset or offset (ie, bed or wake time), total sleep time (ie, sleep duration), sleep efficiency, and the number of minutes awake after sleep onset. Participants will complete 4 questionnaires: National Institutes of Health (NIH) Patient-Reported Outcomes Measurement Information System (PROMIS) Sleep-Related Impairment, a validated, reliable measure of perceived alertness, sleepiness, and tiredness during waking hours and functional impairments because of sleep problems [89]; NIH PROMIS Sleep Disturbance, a validated, reliable measure of perceived sleep quality or satisfaction and difficulty initiating or maintaining sleep [89]; the Munich Chronotype and Horne-Ostberg Morningness-Eveningness Questionnaires, both reliable, valid assessments of participants’ chronotype and morning or evening preference [90]; and the Positive and Negative Affect Scale, a validated, reliable 20-item measure of positive and negative emotion and mood that yields 2 subscales—a positive and negative score [91]. Sleep improvement may cause mood changes that could affect alcohol outcomes. In the A+SM and A+SM+F conditions, participants will complete the Pittsburgh Sleep Diary, a well-validated assessment of daytime sleep-related behaviors and nocturnal sleep characteristics [92]. Participants will record daytime sleep-related behaviors and nocturnal sleep characteristics of the preceding day. Diaries will include questions about caffeine use and ratings of sleep quality, mood, and sleepiness upon waking.

#### Mechanisms of Sleep Intervention Component Effects

To evaluate potential intervention mechanisms, we will assess several TPB constructs based on a prior TPB growth model of risky drinking in young adults [93]. A possibility is that greater awareness of one’s behaviors through active SM and/or feedback about the association between sleep and alcohol use may alter participants’ beliefs and attitudes about drinking. The Behavioral Intentions Questionnaire includes 2 internally consistent items to assess intentions to engage in risky drinking [94]. Reliable and valid adapted versions of the Global Attitudes Scale [93,95,96] and Subjective Norms Questionnaire [95,97] will be used to assess participants’ overall opinions about heavy alcohol consumption, perceptions of how others view their drinking, and their perceptions of typical drinking among their peers. A reliable and valid adapted version of the Drinking Refusal Self-Efficacy Questionnaire [93,98] and additional items suggested by Azjen [95] will assess participants’ perceptions of being able to control or resist heavy drinking.

It is also possible that improving sleep might have direct effects on cognitive mechanisms linked to alcohol-related risks. We will also administer two computer tasks to test these potential intervention mechanisms. The Stop Signal Task is a reliable, valid computerized task that assesses self-control, specifically the ability to inhibit an inappropriate response. The ability to inhibit responding has been shown to be related to alcohol use and to be sensitive to changes in sleep [70,78]. In the Stop Signal Task, participants are instructed to respond when an *O* signal is present but refrain from responding when an *X* signal immediately follows it. They will complete 300 such trials over a 30-minute session (with a 1 minute break every 10 minutes) at intake and week four. The delay between the two signals (stop-signal delay, SSD) will be initially 200 milliseconds, then increased by 32 milliseconds after each successful trial (making the response inhibition more challenging) and decreased by 32 milliseconds after each unsuccessful trial. This titration should yield a mean success rate of 50% (SD 10%) over the session and estimate the critical SSD corresponding to exactly 50% success. This critical SSD minus the participant’s average reaction time to the *O* signal (go reaction time) at that session defines the amount of additional time the participant required to inhibit the inappropriate response (stop-signal reaction time [SSRT]). Thus, a higher SSRT indicates worse inhibitory abilities. A titration increment of 32 milliseconds was chosen because it yielded the desired 50% (SD 10%) success rate among the 4 pilot participants. The N-Back Task, another reliable, valid computerized task, will be used to assess working memory. Performance is related to alcohol use and is sensitive to changes in sleep [77,91]. In the N-Back Task, participants are presented with a series of letters and instructed to respond when the letter matches the letter presented *N* stimuli before. A series of difficulty levels with *N* values ranging from 1 to 3 will be used. Both tasks will be administered at intake and week four.

#### Intervention Component Feasibility and Acceptability

We will evaluate participant use metrics to determine intervention component feasibility (ie, diary and biosensor adherence, use of sleep hygiene tips during treatment and follow-up). At week 2, all participants will complete an end-of-treatment evaluation form. We will also interview participants in A+SM+F to evaluate their reactions to and preferences for sleep or alcohol data monitoring and feedback.

### Statistical Analyses

Statistical analyses will use an intent to treat approach and mixed models, both gold standards (along with multiple imputation) for handling outcome variable missing data in longitudinal studies. For the analyses of primary and secondary outcomes, a type I error of 5% (two-sided) will be used to test for statistical significance using SAS V9.4. Exploratory analyses will be adjusted for multiple testing using the Bonferroni correction. Data will be examined for conformity to the normal distribution, and transformation or nonparametric methods will be used if necessary.

The goal of the primary aim is to examine the effect of the intervention condition over time on total drinks consumed over weeks 4 to 12, controlling for baseline total drinks. For this analysis, we will evaluate changes in scores using a mixed model repeated measures analysis with condition and sex as between-subject factors and time as a within-subject factor. We will also test changes in secondary alcohol outcomes: drinks per day, drinks per drinking day, and alcohol-related consequences, controlling for the corresponding baseline measure alcohol outcomes, which we will adjust for multiple comparisons. Using all repeated measures on individuals in the context of a mixed model will allow us to assess temporal patterns of change over time and to use all available data on an individual. This approach therefore helps to avoid imputing missing data. The mixed model allows us to obtain unbiased and efficient estimates of change over time and between-group differences. Within the mixed model, our primary hypothesis is the change from baseline to end-point, and we will perform focused comparisons to assess these effects. A mixed model will account for the correlation between alcohol outcomes measured in the same individual. We will select the best-fitting variance-covariance structure using the Schwartz-Bayesian information criterion. Time will be considered as a categorical factor, but we will also evaluate whether alcohol outcomes change linearly by condition over time. We will evaluate the alcohol outcomes for normality assumptions. If an outcome is not normally distributed, we have several options, including applying transformations or utilizing alternative methods (eg, generalized linear mixed models, resampling, nonparametric tests). For drinking outcomes that may be best modeled as count data, we can use mixed models using Poisson and negative binomial generalized linear mixed models.

A secondary aim is to examine the effect of condition on sleep quality ratings over time, controlling for baseline ratings. We will use a mixed model repeated measures analysis as described above. We will also test changes in secondary sleep outcomes: ratings of sleep-related impairment and quantitative sleep outcomes (ie, duration, efficiency, number of minutes awake after sleep onset, bed or wake times), controlling for baseline responses. We will evaluate the sleep outcomes for normality assumptions. If an outcome is not normally distributed, we will apply transformations or utilize alternative methods.

Another secondary aim is to summarize participants’ acceptability ratings of mobile sleep hygiene advice, sleep or alcohol diary SM, sleep or alcohol biosensor use, and personalized sleep or alcohol data feedback using descriptive statistics. We anticipate that the A+SM+F condition will yield the highest acceptability ratings for all 3 conditions. A review of participants’ reactions to personalized feedback will provide insight into what types of feedback and tailored health tips are feasible and useful for heavy-drinking young adults.

As an exploratory aim, we will evaluate improvements in TPB constructs (ie, drinking intentions and attitudes, perceived drinking norms, perceived control over drinking, and cognitive task performance) over time as mechanisms of condition effects on total drinks at month 3. We will calculate individual slope change estimates for sleep quality and TPB constructs and then evaluate these slope estimates as potential mechanisms using the SAS macro outlined by Valeri and Vanderweele [99]. This method allows for independent variable X mediator interactions and is suitable for continuous and count outcomes (ie, total drinks). Given the smaller sample size for this exploratory research, we will evaluate correlations between sleep quality and TPB construct slope estimates rather than structural equation modeling to model potential complex pathways among conditions, TPB constructs, and drinking. We will also characterize the daily variations in the sleep diary and biosensor data and potential dynamic relationships between them and daily diary or biosensor drinking data using methods for longitudinally intensive data (eg, time-varying effects models) [100].

Sample size estimates were based on enrolling a sufficient number of participants to ensure adequate power to detect a clinically significant medium effect in total drinks consumed over time, controlling for total drinks consumed at baseline, among the three conditions, which is lower than the medium-to-large effect sizes for alcohol-related outcomes observed in our pilot study. Sample size estimates were obtained under the following assumptions: 80% power, a two-sided .05 significance level, medium effect size for the between-group difference (Cohen *f*=0.3; Cohen *d*=0.6 for the comparison of the personalized feedback condition with the other two conditions), one binary stratification variable (ie, sex), and 10% drop out. On the basis of these metrics, we estimated that a total sample size of 120 individuals would be required to complete the study.

## Results

This project was funded by the NIH in May 2018, and data collection began in December 2018 following institutional review board (IRB) approval. In December 2018, IRB approval was obtained and data collection began. We first enrolled 5 pilot participants to finalize the study procedures. After completing the pilot, we enrolled 110 individuals in the randomized controlled trial. The completion rates for the 2-week intervention phase (107/110, 97.3%) and 12-week follow-up (92/98, 94%) were high. Similarly, adherence to monitoring activities was high: diaries (819/882, 92.9% possible diaries) and nighttime Actiwatch use (1119/1176, 95.15% possible assessment points). Data collection for the final 10 participants is expected to be concluded in early 2021.

## Discussion

### Conclusions and Future Directions

We hypothesize that a multimodal, mobile sleep intervention will reduce drinking and alcohol-related harm in heavy-drinking young adults. We anticipate that sleep or alcohol diary SM and/or personalized feedback about sleep or alcohol data will be the most effective sleep intervention techniques for this purpose. We will use the results to finalize the sleep intervention for future testing. We will then evaluate this sleep intervention against standard alcohol interventions for young adults in a phase II randomized controlled trial. The rich database of objective and subjective sleep and alcohol data will enable us to explore relationships among these variables to inform our understanding of the role of sleep in young adult AUD risk.

## Appendix

Multimedia Appendix 1 NIH grant peer-review documentation.

## Abbreviations

AUD: alcohol use disorder
AUDIT-C: Alcohol Use Disorders Identification Test–Consumption
BAC: blood alcohol concentration
DSM-V: Diagnostic and Statistical Manual of Mental Disorders, fifth edition
IRB: institutional review board
NIH: National Institutes of Health
PROMIS: Patient-Reported Outcomes Measurement Information System
SCRAM: secure continuous remote alcohol monitor
SM: self-monitoring
SSD: stop-signal delay
SSRT: stop-signal reaction time
TAC: transdermal alcohol concentration
TPB: Theory of Planned Behavior
