# Supplementary material for: A Multimodal Mobile Sleep Intervention for Young Adults Engaged in Risky Drinking: Protocol for a Randomized Controlled Trial
Source: JMIR Res Protoc. 2021 Feb 26;10(2):e26557. doi: 10.2196/26557 (PMC7954653; doi:10.2196/26557)
Supplement: Multimedia Appendix 1 [file resprot_v10i2e26557_app1.pdf]

SUMMARY STATEMENT
(Privileged Communication)

PROGRAM CONTACT:
MARIELA SHIRLEY
301-402-9389
shirleym@mail.nih.gov

Release Date: 11/17/2017
Revised Date:

Application Number: 1 R34 AA026021-01A1

Principal Investigator

FUCITO, LISA M

Applicant Organization: YALE UNIVERSITY

Review Group: AA-3 (03)
Clinical, Treatment and Health Services Research Review Subcommittee

Meeting Date: 11/01/2017
Council: JAN 2018
Requested Start: 04/01/2018

RFA/PA: PA16-073
PCC: AP C
Dual PCC: CC/ALA
Dual IC(s): DA

Project Title: DEVELOPMENT OF A MULTIMODAL MOBILE SLEEP INTERVENTION USING WEARABLE TECHNOLOGY TO REDUCE HEAVY DRINKING IN YOUNG ADULTS
SRG Action: Impact Score:12
Next Steps: Visit https://grants.nih.gov/grants/next_steps.htm
Human Subjects: 30-Human subjects involved - Certified, no SRG concerns
Animal Subjects: 10-No live vertebrate animals involved for competing appl.
Gender: 1A-Both genders, scientifically acceptable
Minority: 1A-Minorities and non-minorities, scientifically acceptable
Children: 3A-No children included, scientifically acceptable
Clinical Research - not NIH-defined Phase III Trial

Project Year	Direct Costs Requested	Estimated Total Cost
1	150,000	251,250
2	150,000	251,250
3	150,000	251,250
TOTAL	450,000	753,750

ADMINISTRATIVE BUDGET NOTE: The budget shown is the requested budget and has not been adjusted to reflect any recommendations made by reviewers. If an award is planned, the costs will be calculated by Institute grants management staff based on the recommendations outlined below in the COMMITTEE BUDGET RECOMMENDATIONS section.

1R34AA026021-01A1 FUCITO, LISA

RESUME AND SUMMARY OF DISCUSSION: This is an amended R34 application from an excellent investigator to develop and test a mobile web-based sleep and alcohol self-monitoring intervention for young adults. The proposed studies build on strong pilot data, and target drinking indirectly by addressing sleep hygiene. The research design is sound and employs novel wearable biosensors to objectively assess alcohol use and sleep. The research team and environment are strong and well equipped to carry out the proposed research. The applicant is highly responsive to prior critiques and has satisfactorily addressed all prior concerns. A minor remaining concern - whether the two-week intervention monitoring period is adequate to observe alcohol effects on sleep, is easily addressable. Overall, there was a strong enthusiasm for this application.

DESCRIPTION (provided by applicant): Alcohol use disorder (AUD) onset peaks during young adulthood. Young adults report more frequent, heavier alcohol use than older adults and experience substantial negative consequences (e.g., accidental injury) and risk of developing a chronic AUD. Current young adult alcohol intervention strategies, however, have modest effects. To address this gap, we propose to develop a novel, mobile sleep intervention for heavy-drinking young adults. Sleep may be a useful treatment target for this population for several reasons. Poor sleep is common among young adults who drink heavily and an AUD risk factor. Young adults are also interested in information to help them sleep better. Further, as a standard practice, behavioral sleep interventions address alcohol use and may provide a gateway for intervening on alcohol use. We conducted a preliminary test of a mobile sleep intervention in 42 heavy-drinking young adults. The primary components were: (1) brief, web- based sleep hygiene advice including the standard advice to moderate drinking for better sleep and (2) daily web-based sleep and alcohol self-monitoring plus wearing a sleep/wake activity tracker daily. Our results demonstrated promising effects on drinking and sleep. The next phase of intervention development involves testing our intervention components compared to matched control conditions and whether a new component suggested by participants in post-treatment interviews further improves outcomes. Specifically, participants indicated a preference for personalized feedback about their sleep diary and tracker data and the connections with alcohol use. Additionally, exciting new alcohol biosensor technology has emerged that allows continuous tracking of blood alcohol level and provides an objective measure of participant alcohol consumption. Through this technology we can provide personalized alcohol-sleep interaction feedback using both objective sleep/alcohol trackers and sleep/alcohol diary data. The current proposal will develop and test a mobile sleep/alcohol self-monitoring + sleep/alcohol data feedback intervention in 120 heavy-drinking young adults. All participants will wear sleep and alcohol trackers daily. The primary intervention will include: (1) web-based sleep hygiene advice + sleep/alcohol diary self-monitoring + sleep/alcohol data feedback. This condition (n=60) will be compared to the matched control conditions that only include these components: (1) web-based sleep hygiene advice (n=30) or (2) web-based sleep hygiene advice + sleep/alcohol diary self-monitoring (n=30). The primary objective is to evaluate sleep intervention component feasibility, acceptability, and preliminary efficacy on alcohol outcomes to inform a Stage II randomized trial comparing the final sleep intervention with a standard alcohol intervention. This study is in line with NIAAA’s strategic interest to identify novel targets to improve alcohol prevention and intervention efforts for young people. Ultimately, this research could result in an efficacious, low-cost intervention that has broad population reach through the use of technology and a substantial public health impact by reducing AUD risk at a crucial developmental stage.

PUBLIC HEALTH RELEVANCE

Targeting sleep concerns may be a novel strategy for reducing increased risk of alcohol use disorders (AUDs) in young adults. The results of this project will lead to the development of a well-specified, novel, highly transportable, mobile sleep intervention that could have a great impact on population health.

CRITIQUE 1

Significance: 2
Investigator(s): 1
Innovation: 2
Approach: 1
Environment: 1

Overall Impact:

This revised R34 application seeks to develop and test a mobile sleep and alcohol self-monitoring intervention for young adults. Specifically, a sample of 120 heavy drinking young adults will wear sleep and alcohol trackers daily and will be assigned to one of three conditions: (1) web-based sleep hygiene advice + sleep/alcohol diary self-monitoring + sleep/alcohol data feedback (A + SM + F; n=60), (2) web-based sleep hygiene advice (A; n=30), or (3) web-based sleep hygiene advice + sleep/alcohol diary self-monitoring (A + SF; n=30). The primary objective is to evaluate sleep intervention component feasibility, acceptability, and preliminary efficacy on alcohol outcomes to inform a Stage II randomized trial comparing the final sleep intervention with a standard alcohol intervention. The investigative team was very responsive to the initial critiques and have revised the application to clarify its broader generalizability (i.e., not limited to college students) as well as the overall framework which consists of identifying sleep as a “hook” into alcohol intervention. The distinction between sleep and alcohol problems co-occurrence in late versus early stages of drinking is also well taken. Overall there is considerable enthusiasm for this proposal as well as for the investigator.

1. Significance:

Strengths

- Addressing alcohol use during young adulthood is a highly significant research area and attending to sleep may provide a useful “hook” into alcohol treatment
- The proposal fits in nicely within the NIAAA strategic plan and the opportunities for combining wearable technology with evidence-based interventions are vast

Weaknesses

- While sleep concerns are a “hook” into treatment, the wide scale application of the treatment may be somewhat limited (though not a significant concern)

2. Investigator(s):

Strengths

- Excellent team led by Dr. Fucito who has recently completed a K23 award
- Dr. O’Malley is an outstanding mentor and has a track record of supporting Dr. Fucito. She also has ample experience with interventions in heavy drinking young adults.

Weaknesses

- The applicant’s K23 was focused on heavy drinking smokers and the proposed study is not a direct extension of her K23 work (although not a significant drawback)

3. Innovation:

Strengths

- Combining sleep and alcohol intervention in young adults is novel given that most of the literature is on older adults.
- The proposed wearable technology is also innovative, particularly in combination with well-established interventions such as personalized feedback.

Weaknesses

- None noted

4. Approach:

Strengths

- Excellent RCT design with well-justified methods
- Thoughtful data analytic plan, including a discussion of the use of small sample pilot data to generate effect sizes (which was aptly not undertaken by the team based on the literature)
- Excellent response to previous critiques clarifying methods such as the use of go/no-go task and its practice effects, managing wearable technology, and broadening scope of work to all young adults (not college drinkers)
- Specific plans for an R01 that would stem from this R34 are carefully laid out and include two proposals focused on a large scale RCT as well as the development of large scale wearable technology intervention.

Weaknesses

- While all studies have pros-cons in their design, the currently proposed study appears to effectively balance its design considerations to properly test the study aims. As such, no significant weaknesses to the approach section are noted.

5. Environment:

Strengths

- Excellent environment at Yale where the PI has been successful in carrying out her research for the past 10 years.
- Resources for intervention studies in heavy drinking young adults are plentiful at Yale and its surrounding areas.

Weaknesses

- None noted.

Protections for Human Subjects:

Adequate

Data and Safety Monitoring Plan (Applicable for Clinical Trials Only):

Acceptable

Inclusion of Women, Minorities and Children:

- Sex/Gender: Distribution justified scientifically
- Race/Ethnicity: Distribution justified scientifically
- For NIH-Defined Phase III trials, Plans for valid design and analysis: N/A

- Inclusion/Exclusion of Children under 18: Distribution justified scientifically

Budget and Period of Support:

Recommend as requested

CRITIQUE 2

Significance: 2
Investigator(s): 1
Innovation: 2
Approach: 2
Environment: 1

Overall Impact

Current alcohol intervention strategies in young adults have modest effects, and the proposed revised R34 study proposes to develop a novel, mobile sleep intervention for heavy- drinking young adults. Poor sleep is common among young adults and an AUD risk factor, and young adults are more interested in interventions to promote sleep than in interventions to reduce drinking. Although sleep interventions have not reduced drinking in more severe adult populations, they may have potential as an indirect way of targeting drinking in young adult drinkers who are not motivated to complete alcohol-focused interventions. 120 heavy-drinking young adults with sleep difficulties will be randomly assigned to one of 3 groups: (1) - web-based sleep hygiene (N = 30), (2)- web-based sleep-hygiene plus sleep diary and alcohol self-monitoring (30), and (3) - web-based sleep hygiene, plus sleep diary and alcohol self-monitoring (N = 60). All participants will wear sleep and alcohol trackers daily. The primary objective is to evaluate sleep intervention component feasibility, acceptability, and preliminary efficacy on alcohol outcomes to inform a STTR application with a technology partner and a Stage II randomized trial. This proposal has many strengths, including the strong pilot data, novelty and potential significance of targeting drinking indirectly through a more palatable focus on sleep (both are potentially important health outcomes), the careful assessment approach including the state-of-the-science alco-tracker measure of alcohol use and sleep, and the strong team and environment. Improving sleep may reduce risk-taking in young adults, such as heavy drinking while circumventing the resistance associated with traditional alcohol interventions. This revision has highly responsive and addressed most limitations identified in the initial review. One concern is that the two-week intervention monitoring period may not be long enough to record multiple drinking episodes in order for participants to observe alcohol effects on sleep. However, overall this is a very strong application.

1. Significance:

Strengths

- Current alcohol intervention strategies in young adults have modest effects and sleep may be a useful adjunctive treatment target for this population because poor sleep is common among young adults and an AUD risk factor, and young adults are also interested in information to help them sleep better.
- Behavioral sleep interventions address alcohol use and may provide a gateway (motivational hook) for intervening on alcohol use avoiding the stigma and low motivation that generally results in very low utilization of available brief alcohol interventions.

- Enhancing sleep may improve health and social functioning independent of change in alcohol misuse, and improved sleep may bolster self-control which may also contribute to alcohol reductions.
- Analyses could determine if self-control is mechanism of change
- Alco-track monitoring of sleep and alcohol use could generate data that extends knowledge of the relations between sleep and drinking among young adults.
- The intervention appears to be relatively inexpensive and not highly burdensome which increases the potential for dissemination. Moreover, enhancing sleep has benefits on overall wellness and learning and is therefore consistent with overall university efforts to promote wellness.

Weaknesses

- the proposed research would only benefit those who have sleep difficulties and a desire to improve their sleep
- Many college student risky drinkers drink only 1-2 times per week and compensate by sleeping late the day after and may experience very little meaningful sleep impairment that is directly related to drinking. The risks associated with college drinking are generally due to occasional episodes of extreme drinking or decision such as drinking and driving, rather than regular patterns of heavy drinking that might be most related to poor sleep.
- There are efficacious brief interventions for college drinking, and although there is a need to enhance effect sizes, it is not clear if this proposed intervention would generate larger effects than current BMIs, or if it would complement/extend BMI effects.

2. Investigator(s):

Strengths

- The PI is a highly productive Associate Research Scientist with some expertise in the role of sleep in addiction who is supported by early stage investigator who has been supported by an NIAAA K23 that includes a focus on sleep and addiction. She has collaborated extensively with the investigative team which includes all the requisite experience to successfully complete this study.

Weaknesses

- None noted

3. Innovation:

Strengths

- Addressing alcohol misuse indirectly via a sleep enhancement intervention is innovative
- Remote objective assessment of sleep and alcohol use (alco-tracker) is innovative
- Direct test of self-control as mechanism of behavior change is also novel
- The study provides the first systematic, controlled test of the effects of various sleep intervention techniques on alcohol use and alcohol-related consequences in heavy-drinking young adults

Weaknesses

- Sleep interventions have been attempted with adult heavy drinking samples without clear benefits. However, it is possible less severe young adult drinkers will show better response.

4. Approach:

Strengths

- Strong RCT design, appropriate follow-up intervals, detailed recruitment plan with strong track record of successful recruitment for similar research
- Design controls for assessment reactivity (comparing advice only vs. advice plus monitoring) while also allowing for a moderately powered test of active treatment (n = 60) vs 2 control conditions (n = 60)
- Very strong and comprehensive assessment plan, including alco-tracker assessment of sleep and drinking
- Clear and compelling analytic plan
- Pilot data recently published in ACER - 42 heavy-drinking young adults completed a (1) brief, web-based sleep hygiene advice including the standard advice to moderate drinking for better sleep and (2) daily web-based sleep self-monitoring (including drinks before bedtime) plus wearing a sleep/wake activity tracker daily. Results demonstrated feasibility and promising effects on drinking and sleep.

Weaknesses

- The two-week intervention monitoring period may not be long enough to record multiple drinking episodes in order observe the objective effects on sleep given that the inclusion criteria is 4 heavy drinking episodes in past 30 days. Given that study entry and the effects of monitoring may further reduce drinking, many participants may only experience 1-2 drinking episodes during 2-week period. The inclusion criteria should map on better to the intervention period (i.e., require minimum frequency of past 2-week drinking or include 4 weeks of monitoring to provide an adequate period for participants to learn more about the impact of drinking on sleep). The pilot trial included 4 weeks of monitoring.

5. Environment:

Strengths

- Yale provides a very strong research environment and all resources necessary to conduct the proposed research. The team has conducted relevant pilot work and includes expertise in alcohol and sleep.

Weaknesses

- None

Protections for Human Subjects:

Adequate protections

- Data and Safety Monitoring Plan (Applicable for Clinical Trials Only):

  Acceptable

  - An independent DSMB has been formed for the trial

Inclusion of Women, Minorities and Children:

- Sex/Gender: Distribution justified scientifically
- Race/Ethnicity: Distribution justified scientifically
- For NIH-Defined Phase III trials, Plans for valid design and analysis: Not applicable

- Inclusion/Exclusion of Children under 18: Excluding ages <18; justified scientifically

Biohazards:
Not Applicable (No Biohazards)

Resubmission:

- The resubmission is highly responsive to the original reviews.

Applications from Foreign Organizations:
Not Applicable (No Foreign Organizations)

Select Agents:
Not Applicable (No Select Agents)

Resource Sharing Plans:
Adequate

Authentication of Key Biological and/or Chemical Resources:
Not Applicable (No Relevant Resources)

Budget and Period of Support:
Recommended budget modifications or possible overlap identified:

CRITIQUE 3

Significance: 1
Investigator(s): 1
Innovation: 2
Approach: 2
Environment: 1

Overall Impact:
This study aims to provide compare three arms of a sleep intervention (sleep hygiene advice, advice + monitoring, advice + monitoring + feedback) aimed at improving sleep and reducing alcohol use in young adults. Building from pilot research that demonstrated feasibility, this study aims to discern whether sleep and alcohol tracking and/or feedback enhances the effects of an advice-alone intervention on sleep and heavy alcohol use. Interestingly, selected young adults will come from a population of those with sleep problems, not with those with concerns about alcohol use. This has the potential to engage far more young adults in alcohol treatment than an alcohol intervention alone. The potential impact of a sleep intervention with spillover benefits to alcohol use in young adults could be quite large, particularly if the intervention could be easily implementable in common health care settings. The use of objective measures of alcohol use and sleep is innovative, particularly because it allows measurement without any possibility of inadvertent self-monitoring effects (as compared to self-report), thus strengthening the inferences that can be drawn between the Advice alone and Advice +

Monitoring condition. Repeated in-vivo assessments of sleep and alcohol use also increase reliability in the estimation of between and within person associations, and provides an innovative means to test the mechanism and temporal dynamics of intervention effects. In short, this is an innovative proposal that has potential to broadly engage young adults in alcohol intervention who may not have previously been interested in behavior change.

1. Significance:

Strengths

- Poor sleep is a common complaint among heavy drinking young adults, and is a risk factor for AUD, and poor sleep and heavy drinking may dynamically interact to raise and maintain risk for both over time. Interventions targeting sleep may be a means to target heavy drinking young adults without the stigma associated with seeking treatment for alcohol use. As such, this kind of “foot in the door” intervention could be very promising.

Weaknesses

- None noted.

2. Investigator(s):

Strengths

- The project is an extension of the PI’s training from the K-23 award, mixing sleep research, intervention and additions.
- The investigative team is strong, with a history of collaboration and expertise in the appropriate content domains.

Weaknesses

- None noted.

3. Innovation:

Strengths

- Targeting alternative strategies to engage young adults in alcohol treatment is innovative.

Weaknesses

- None noted.

4. Approach:

Strengths

- Sleep and alcohol use will be measured with a mix of passive and active measurements that have good evidence of reliability. Passive measurement of sleep will be especially important for the advice alone condition, to eliminate the influence of self-monitoring on sleep behavior.
- Pilot data suggested a high level of interest in the sleep intervention, and also suggested that following recruitment, all participants exhibited improved sleep. The proposed study will attempt to disentangle these findings by including a monitoring only condition, without a sleep diary, which will allow the investigators to test whether improvements were due only to self-monitoring, regression to the mean, or the active proposed treatment.

- The explanatory mechanisms (theory of planned behavior constructs) are more clearly specified in the revision.
- The investigators have reduced the number of assessments of the Go/No-Go task, reducing the possibility of reactivity.
- The investigators have clarified how non-normal and count data will be handled (using a variety of appropriate approaches including resampling and generalized mixed linear models), as well as how missing data will be treated.

Weaknesses

- The statistical analysis is greatly improved, but the power section only provides an omnibus power analysis, and no description of how power was computed. It's not clear that the power analysis modeled the power of the proposed analysis to detect the main effects of treatment or the other proposed aims of the study. It is likely that the study is adequately powered, and the analyses are more thoroughly described, but it needs to be clear that the power analysis followed the analytic description.
- This remains a minor comment that does not detect from the overall strength of the application. I remain skeptical of the Go/No-Go task representing an objective measure of self-control (for details of the psychometric problems with behavioral measures of impulse control, see reviews and meta-analyses by Cyders, Duckworth, Sharma, among others). Behavioral tasks tend to not correlate with one another, nor with self-report, and outcomes are so inconsistently reported in the literature it is hard to believe that they do anything but capitalize on chance. The authors may consider dropping the baseline assessment to entirely rule out practice effects. Per the CONSORT guidelines for RCTs, so long as randomization was successful, one must assume any differences across conditions arose due entirely to chance. Thus, assessing the Go/No-Go at baseline provides little benefit, and runs the risk of inducing some practice effect.

5. Environment:

Strengths

- Excellent.

Weaknesses

- None noted.

Protections for Human Subjects:

Acceptable Risks and/or Adequate Protections

- Appropriate

Data and Safety Monitoring Plan (Applicable for Clinical Trials Only):

Not Applicable (No Clinical Trials)

Inclusion of Women, Minorities and Children:

- Sex/Gender: Distribution justified scientifically
- Race/Ethnicity: Distribution justified scientifically
- For NIH-Defined Phase III trials, Plans for valid design and analysis: Not applicable
- Inclusion/Exclusion of Children under 18: Excluding ages <18; justified scientifically

Vertebrate Animals:
Not Applicable (No Vertebrate Animals)

Biohazards:
Not Applicable (No Biohazards)

Resubmission:

- The investigators have been generally responsive to the reviews and have presented an improved application

Applications from Foreign Organizations:
Not Applicable (No Foreign Organizations)

Select Agents:
Not Applicable (No Select Agents)

Resource Sharing Plans:
Not Applicable (No Relevant Resources)

Authentication of Key Biological and/or Chemical Resources:
Not Applicable (No Relevant Resources)

Budget and Period of Support:
Recommend as Requested

THE FOLLOWING SECTIONS WERE PREPARED BY THE SCIENTIFIC REVIEW OFFICER TO SUMMARIZE THE OUTCOME OF DISCUSSIONS OF THE REVIEW COMMITTEE, OR REVIEWERS' WRITTEN CRITIQUES, ON THE FOLLOWING ISSUES:

PROTECTION OF HUMAN SUBJECTS: ACCEPTABLE

INCLUSION OF WOMEN PLAN: ACCEPTABLE

INCLUSION OF MINORITIES PLAN: ACCEPTABLE

INCLUSION OF CHILDREN PLAN: ACCEPTABLE

COMMITTEE BUDGET RECOMMENDATIONS: The budget was recommended as requested.

Footnotes for 1 R34 AA026021-01A1; PI Name: FUCITO, LISA M

NIH has modified its policy regarding the receipt of resubmissions (amended applications). See Guide Notice NOT-OD-14-074 at http://grants.nih.gov/grants/guide/notice-files/NOT-OD-14-074.html. The impact/priority score is calculated after discussion of an application by averaging the overall scores (1-9) given by all voting reviewers on the committee and multiplying by 10. The criterion scores are submitted prior to the meeting by the individual reviewers assigned to an application, and are not discussed specifically at the review meeting or calculated into the overall impact score. Some applications also receive a percentile ranking. For details on the review process, see http://grants.nih.gov/grants/peer_review_process.htm#scoring.

MEETING ROSTER

Clinical, Treatment and Health Services Research Review Subcommittee
National Institute on Alcohol Abuse and Alcoholism Initial Review Group
NATIONAL INSTITUTE ON ALCOHOL ABUSE AND ALCOHOLISM
AA-3 (03)
11/01/2017

Notice of NIH Policy to All Applicants: Meeting rosters are provided for information purposes only. Applicant investigators and institutional officials must not communicate directly with study section members about an application before or after the review. Failure to observe this policy will create a serious breach of integrity in the peer review process, and may lead to actions outlined in NOT-OD-14-073 at https://grants.nih.gov/grants/guide/notice-files/NOT-OD-14-073.html and NOT-OD-15-106 at https://grants.nih.gov/grants/guide/notice-files/NOT-OD-15-106.html, including removal of the application from immediate review.

CHAIRPERSON(S)

VOCCI, FRANK J., PHD
PRESIDENT
FRIENDS RESEARCH INSTITUTE, INC.
BALTIMORE, MD 21204

MEMBERS

BRADLEY, KATHARINE ANTHONY, MD, MPH
SENIOR INVESTIGATOR
GROUP HEALTH RESEARCH INSTITUTE
SEATTLE, WA 98101

CHILDS, EMMA, PHD *
ASSOCIATE PROFESSOR
DEPARTMENT OF PSYCHIATRY
UNIVERSITY OF ILLINOIS AT CHICAGO
CHICAGO, IL 60637

CHOI, DOO-SUP, PHD
PROFESSOR OF PHARMACOLOGY AND PSYCHIATRY
DEPARTMENT OF MOLECULAR PHARMACOLOGY
AND EXPERIMENTAL THERAPEUTICS
MAYO CLINIC COLLEGE OF MEDICINE
ROCHESTER, MN 55905

CUNNINGHAM, REBECCA M., MD *
ASSOCIAT CHAIR FOR RESEARCH AND PROFESSOR
DEPARTMENT OF EMERGENCY MEDICINE
UNIVERSITY OF MICHIGAN
ANN ARBOR, MI 48109

FELDSTEIN EWING, SARAH W., PHD
PROFESSOR
DEPARTMENTS OF CHILD & ADOLESCENT PSYCHIATRY
ADOLESCENT BEHAVIORAL HEALTH CLINIC
OREGON HEALTH & SCIENCE UNIVERSITY
PORTLAND, OR 97239

FLEMING, MICHAEL F., MD
PROFESSOR
DEPARTMENTS OF PSYCHIATRY AND BEHAVIORAL SCIENCES
AND FAMILY AND COMMUNITY MEDICINE
FEINBERG SCHOOL OF MEDICINE
NORTHWESTERN UNIVERSITY
CHICAGO, IL 60611

KARNO, MITCHELL P, PHD *
NEUROPSYCHIATRIC INSTITUTE
UCLA INTEGRATED SUBSTANCE ABUSE PROGRAM
LOS ANGELES, CA 90025

KING, KEVIN MICHAEL, PHD
ASSOCIATE PROFESSOR
DEPARTMENT OF PSYCHOLOGY
UNIVERSITY OF WASHINGTON
SEATTLE, WA 98195

MIRANDA, ROBERT JR., PHD
ASSOCIATE PROFESSOR (RESEARCH)
DEPARTMENT OF PSYCHIATRY & HUMAN BEHAVIOR
CENTER FOR ALCOHOL & ADDICTION STUDIES
BROWN UNIVERSITY
PROVIDENCE, RI 02906

MURPHY, JAMES G, PHD
PROFESSOR AND DIRECTOR OF CLINICAL TRAINING
DEPARTMENT OF PSYCHOLOGY
UNIVERSITY OF MEMPHIS
MEMPHIS, TN 38152

RAY, LARA, PHD
ASSOCIATE PROFESSOR
DEPARTMENT OF PSYCHOLOGY
BRAIN RESEARCH INSTITUTE
UNIVERSITY OF CALIFORNIA, LOS ANGELES
LOS ANGELES, CA 90095

SIMPSON, TRACY L., PHD
ASSOCIATE PROFESSOR
DEPARTMENT OF PSYCHIATRY & BEHAVIORAL SCIENCES
SCHOOL OF MEDICINE
UNIVERSITY OF WASHINGTON
SEATTLE, WA 98108

STASIEWICZ, PAUL R, PHD *
SENIOR RESEARCH SCIENTIST
CLINICAL DIRECTOR
RESEARCH INSTITUTE ON ADDICTIONS
THE STATE UNIVERSITY OF NEW YORK AT BUFFALO
BUFFALO, NY 14203

WISDOM, JENNIFER PELT, PHD *
PROFESSOR
DEPARTMENT: OF HEALTH POLICY AND MANAGEMENT
GRADUATE SCHOOL OF PUBLIC HEALTH AND HEALTH
POLICY
CITY UNIVERSITY OF NEW YORK
NEW YORK, NY 10027

<u>SCIENTIFIC REVIEW OFFICER</u>

SRINIVAS, RANGA V., PHD
CHIEF, EXTRAMURAL PROJECT REVIEW BRANCH
EXTRAMURAL PROJECT REVIEW BRANCH
NATIONAL INSTITUTE ON ALCOHOL ABUSE AND
ALCOHOLISM
NATIONAL INSTITUTES OF HEALTH
ROCKVILLE, MD 20855

<u>EXTRAMURAL SUPPORT ASSISTANT</u>

STRINGFIELD, DONNA
EXTRAMURAL ASSISTANT
OFFICE OF EXTRAMURAL ACTIVITIES
NATIONAL INSTITUTES ON ALCHOHOL ABUSE AND
ALCHOHOLISM
NATIONAL INSTITUTES OF HEALTH,
ROCKVILLE, MD 20892

* Temporary Member. For grant applications, temporary members may participate in the entire meeting or may review only selected applications as needed.

Consultants are required to absent themselves from the room during the review of any application if their presence would constitute or appear to constitute a conflict of interest.
